# Vasoactivity of Rucaparib, a PARP-1 Inhibitor, is a Complex Process that Involves Myosin Light Chain Kinase, P2 Receptors, and PARP Itself

**DOI:** 10.1371/journal.pone.0118187

**Published:** 2015-02-17

**Authors:** Cian M. McCrudden, Martin G. O’Rourke, Kim E. Cherry, Hiu-Fung Yuen, Declan O’Rourke, Muhammad Babur, Brian A. Telfer, Huw D. Thomas, Patrick Keane, Thiagarajan Nambirajan, Chris Hagan, Joe M. O’Sullivan, Chris Shaw, Kaye J. Williams, Nicola J. Curtin, David G. Hirst, Tracy Robson

**Affiliations:** 1 School of Pharmacy, Queen’s University Belfast, Belfast, United Kingdom; 2 Centre for Cancer Research and Cell Biology, Queen’s University Belfast, Belfast, United Kingdom; 3 Belfast Health and Social Care Trust, Belfast, United Kingdom; 4 Manchester Pharmacy School, The University of Manchester, Manchester, United Kingdom; 5 Northern Institute for Cancer Research, Newcastle University, Newcastle upon Tyne, United Kingdom; Cinvestav-IPN, MEXICO

## Abstract

Therapeutic inhibition of poly(ADP-ribose) polymerase (PARP), as monotherapy or to supplement the potencies of other agents, is a promising strategy in cancer treatment. We previously reported that the first PARP inhibitor to enter clinical trial, rucaparib (AG014699), induced vasodilation *in vivo* in xenografts, potentiating response to temozolomide. We now report that rucaparib inhibits the activity of the muscle contraction mediator myosin light chain kinase (MLCK) 10-fold more potently than its commercially available inhibitor ML-9. Moreover, rucaparib produces additive relaxation above the maximal degree achievable with ML-9, suggesting that MLCK inhibition is not solely responsible for dilation. Inhibition of nitric oxide synthesis using L-NMMA also failed to impact rucaparib’s activity. Rucaparib contains the nicotinamide pharmacophore, suggesting it may inhibit other NAD+-dependent processes. NAD^+^ exerts P2 purinergic receptor-dependent inhibition of smooth muscle contraction. Indiscriminate blockade of the P2 purinergic receptors with suramin abrogated rucaparib-induced vasodilation in rat arterial tissue without affecting ML-9-evoked dilation, although the specific receptor subtypes responsible have not been unequivocally identified. Furthermore, dorsal window chamber and real time tumor vessel perfusion analyses in PARP-1^-/-^ mice indicate a potential role for PARP in dilation of tumor-recruited vessels. Finally, rucaparib provoked relaxation in 70% of patient-derived tumor-associated vessels. These data provide tantalising evidence of the complexity of the mechanism underlying rucaparib-mediated vasodilation.

## Introduction

Poly (ADP-ribose) polymerase -1 and -2 (PARP-1 and -2) are DNA damage-activated enzymes that participate in multiple DNA repair pathways, including base excision repair [1,2]. Upon binding to DNA breaks, PARP-1/2 ADP-ribosylate themselves, histones H1 and H2B, loosening chromatin and facilitating repair, concomitantly consuming NAD^+^ and releasing nicotinamide [1,2]. PARP-1 or -2 loss or inhibition results in increased sensitivity to DNA alkylating agents, topoisomerase I poisons and ionizing radiation. Attention is now being paid to PARP inhibitors as cancer chemosensitisers [3].

AG14361 (one of a series of tricyclic benzimidazole carboxamide PARP inhibitors [4] is a potent chemo- and radiosensitizer *in vitro* and *in vivo* [5] and inhibits the repair of double strand breaks in DNA, sensitizing cancer cells to ionising radiation [6]. Further development of this series of inhibitors identified AG14447 as a chemosensitizer with ten times the potency of AG14361; the phosphate salt of AG14447 is AG014699, now called rucaparib, which has equivalent potency and improved pharmacological properties [7]. Rucaparib was the first PARP inhibitor tested in cancer patients. Rucaparib displayed encouraging activity in phase I and phase II trials for treatment of metastatic malignant melanoma in combination with temozolomide [8]. There are now several PARP inhibitors in advanced clinical trials, including BMN-673, olaparib, veliparib and niraparib, as well as rucaparib (www.clinicaltrials.gov).

In SW620 xenografts, AG14361 was a more potent chemosensitizer than it was during *in vitro* testing; visualization of the tumor vasculature indicated that this anomaly may be attributable to effects of the drug on tumor blood flow [5]. Rucaparib, like most PARP inhibitors, contains the nicotinamide pharmacophore. Nicotinamide (itself a weak PARP inhibitor) was demonstrated to enhance radiotherapy by increasing tumor perfusion over two decades ago [9]. However, its therapeutic benefit is restricted by its dose-limiting toxicity, emesis, which has been attributed to inhibition of contraction of smooth muscle of the gut, resultant of myosin light chain kinase (MLCK) inhibition [10]. We showed previously that both rucaparib and AG14361 induced relaxation of constricted rat arteries, but only rucaparib inhibited MLCK activity [11]. It is evident that a mechanism more complex than MLCK inhibition is responsible for vasodilation induced by these PARP inhibitors.

The purpose of the current study was to gain a better understanding of the behavior of rucaparib by delineating the mechanism of its vasoactivity using rat arterial tissue and tumor-recruited vascular tissue in wild-type and PARP-1^-/-^ mice. Additionally, we investigated whether freshly excised tumor-associated vascular tissue from patients having undergone nephrectomy for renal cell carcinoma displayed a similar pattern of response to rucaparib. Our results indicate that rucaparib-evoked relaxation of arterial tissue is reliant on MLCK inhibition, is dependent on P2 purinergic receptors, and may involve PARP itself.

## Materials and Methods

### Chemicals and reagents

All chemicals and reagents were from Sigma, Dorset, UK unless otherwise stated. Rucaparib was kindly provided by Pfizer GRD (La Jolla, USA).

### Animals

All animal experiments were carried out in accordance with the Animal (Scientific Procedures) Act 1986 and conformed to the current UKCCCR guidelines. Rat tissue experiments were approved by the Home Office Inspectorate and by the Animal Welfare and Ethics Review Body at Queen’s University, Belfast. Mouse experiments were approved by the Home Office Inspectorate, and the Local Ethical Review Process of The University of Manchester and the Institutional Animal Welfare Committee at Newcastle University. All experiments performed complied with Animal Research: Reporting of *In Vivo* Experiments (ARRIVE) guidelines; for S1 ARRIVE Guidelines Checklist, please see Supporting Information.

Mice were bred in-house and maintained using the highest possible standard of care, and priority was given to their welfare. Any mice identified to be suffering were immediately sacrificed. Rats were purchased from Harlan (UK). Anesthesia of mice was by isoflurane. All animals were sacrificed by CO_2_ asphyxiation.

### Rat tail artery preparation

Tissue preparation was as previously reported [11]. Briefly, male albino Wistar rats (6–12 weeks old) were killed by CO_2_-asphyxiation, and death confirmed by cervical dislocation. The tail was removed from the animal at its most proximal point. The tail artery on the ventral side was identified in the vascular bed, was bathed in ice-cold Krebs’ solution (118 mM NaCl, 4.7 mM KCl, 25 mM NaHCO_3_, 1.15 mM NaH_2_PO_4_, 2.5 mM CaCl_2_, 1.1 mM MgCl_2_, 5.6 mM glucose), and a 2 cm long cannula (1 mm in diameter) was introduced into the artery, and the artery slid over it until an insertion overlap of approximately 5 mm was achieved. The artery was secured to the cannula using double-knotted thread. A length of artery approximately 10 mm beyond the end of the cannula was freed from its vascular bed and cut.

The cannulated artery was connected to an internal/external perfusion apparatus and perfused with oxygenated Krebs’ (95% O_2_/5% CO_2_, BOC, Manchester, UK) at 37°C, slowly at first (0.25 ml/min), increasing to a maximum of 2 ml/min. The artery segments were then allowed to equilibrate for 1 h, before responsiveness was confirmed by constriction with 10 μM phenylephrine (PE).

Arterial constriction or dilation was detected as an increase or decrease in pressure detected by transducers (monitored by water displacement) connected to a MacLab system (AD Instruments Pty Ltd., Australia).

### Rat aorta preparation

The rats used and method of sacrifice were as above. The aorta was removed, fibroadipose tissue was discarded, and the explanted aorta placed in fresh ice-cold oxygenated Krebs’ solution. The aorta was cut into rings 2–3 mm thick, and rings were placed on to the pins of an isometric force transducer and lowered into a tissue bath containing Krebs’ at 37°C, with Krebs’ flowing through at a rate of 2 ml per minute. In this system, one pin is stationary, while the second pin is free to move vertically and is used to apply tension to the piece of tissue. Tension was gradually applied to the aortic ring until a preload of approximately 0.5 g was reached. Constriction of the aortic ring was produced by perfusing the organ bath with 10 μM PE. Aortic constriction or relaxation was detected by an increase or decrease in force detected by the transducers connected to a MacLab system (AD Instruments Pty Ltd., Australia).

### Pharmacological assessment of rucaparib

Arterial tissues were constricted using 10 μM PE. Following this, the tissues were exposed to perfusate that contained 10 μM PE and the relevant concentration of rucaparib (Pfizer GRD, La Jolla, California), which in most cases was in the 10 ρM to 100 μM range. The degree of relaxation elicited by rucaparib treatment was expressed as a percentage of the magnitude of constriction that was observed following 10 μM PE treatment.

The role of nitric oxide (NO˙) in rucaparib-evoked vasodilation was assessed by pre-treating arterial segments from both sites with increasing concentrations (10 nM—100 μM) of the nitric oxide synthase (NOS) inhibitor *N*_ω_-methyl-L-arginine acetate salt (L-NMMA) before challenge with a combination of the relevant concentration of L-NMMA plus 100 μM rucaparib. The presence of an intact endothelium was demonstrated by acetylcholine-evoked relaxation of PE-constricted artery tissues.

To investigate the effects of direct inhibition of MLCK on arterial smooth muscle contraction, tissue segments were constricted using 10 μM PE as previously described, before being treated with a combination of PE and a range of concentrations of ML-9 (a potent MLCK inhibitor), and a concentration response curve was constructed. The maximally-relaxant concentration of ML-9 was then used in combination with 100 μM rucaparib and the degree of relaxation was expressed as a percentage of the magnitude of constriction that was observed following 10 μM PE treatment. Significance of differences between treatments was assessed using Student’s *t*-test.

P2 receptor involvement in rucaparib-evoked dilation was determined by succeeding the initial 10 μM PE with perfusate containing 10 μM PE plus suramin (100 pM—10 μM), perfusing for 20 min to allow equilibration, before perfusing with PE, suramin and 100 μM or 500 μM rucaparib (tail artery and aorta respectively). These protocols were duplicated to address the involvement of P2 receptors in nicotinamide- and ML-9-evoked dilation. Specific P2 receptors were antagonised using oxidised ATP (P2X_7_; 100 μM) Brilliant Blue G (P2X_4_, P2X_7_; 1 μM) and MRS-2179 (P2Y_1_; 10 μM).

### Measurement of the potency of rucaparib’s inhibition of MLCK

Kinase activity analysis was performed as reported previously, using the Millipore IC_50_ *Profiler* Express service [11]. To determine the relative potency of rucaparib, ML-9 was incorporated into the assay. 5 mM samples of rucaparib and ML-9 were supplied to the Drug Discovery Service department at Millipore (Dundee, Scotland). 10-point, ½ logarithmic dilution series were generated, and MLCK phosphorylation of the peptide substrate (KKLNRTLSFAEPG) was analysed in duplicate. The relative activity of MLCK following treatment with the relevant agent was then expressed as a percentage of the activity of the kinase in control (1% (v/v) DMSO).

### Role of PARP in vasoactivity of rucaparib

These studies were undertaken to determine the responsibility of PARP-1 in rucaparib’s vasoactivity, and to determine whether rucaparib impacts the tone of mouse microvessels *in vivo* as it does rat macrovessels *ex vivo*. Vessel mismatch studies were used as an *ex vivo* model of tumor vessel perfusion as previously described [11]. PARP-1^-/-^ mice (C57BL/6 background strain) were obtained from the de Murcia group [12], and were maintained in Newcastle for more than 10 generations. Mice used in vessel mismatch and dorsal window chamber experiments were 8–10 weeks old. Wild-type (WT) mice were not littermates of the PARP-1^-/-^ mice, but rather products of a WT C57BL/6 colony.

For vessel mismatch assessment, once B16 (European Collection of Cell Cultures) tumors grown subcutaneously on the flank of C57BL/6 WT or PARP-1^-/-^ mice achieved a diameter of 10 mm, rucaparib (1 mg/kg—a concentration sufficient to sensitize to temozolomide [11]) was administered i.p, followed 30 min later by Hoechst 33342 (i.v., 15 mg/kg, dissolved in PBS) and a further 20 min later by carbocyanine (i.v., 1 mg/kg, dissolved in 75% dimethyl sulphoxide). 5 min following carbocyanine delivery, mice were sacrificed, tumors were excised and rapidly frozen. We previously reported that plasma levels of rucaparib were undetectable 4 h post-administration of rucaparib to Capan-1 tumor-bearing mice, while intratumoral levels persisted for ≥ 48 h, and PARP-1 inhibition persisted for ≥ 7 days [13]. Owing to rucaparib’s rapid delivery and prolonged activity, we chose to analyze vessel perfusion shortly following administration. A Nikon Eclipse E800 microscope was used to analyze the Hoechst 33342 and carbocyanine (excitation wavelengths, 340–380 nm and 450–490 nm; emission wavelengths, 480 nm and 510 nm, for Hoechst and carbocyanine, respectively) content of vessels in 10 μm tumor sections.

Real time dorsal window chamber analysis of tumor vessel perfusion was as previously described [14]. Briefly, chambers implanted in C57BL/6 WT or PARP-1^-/-^ mice were inoculated with 30 μl of B16 cells at 5x10^7^/ml. When tumors were established, intravital microscopy was used to assess vascular parameters in anesthetized mice. Images were captured in both bright field and fluorescence before and after administration of rucaparib (ip 1 mg/kg) using a Nikon Eclipse E800. Real time tumor perfusion was assessed by quantification of the accumulation of BSA labeled with Alexa-Fluoro-647 (BSA-647; excitation wavelength 647 nm, 1 mg/mL in sterile saline; Molecular Probes; Invitrogen) that was delivered i.v. (100 μl/mouse) before rucaparib treatment. A Metamorph analysis system was used to quantify fluorescence emission at 668 nm. Significance of differences between treatments was assessed using Student’s *t*-test.

### Provision of human tumor-associated vascular tissue

Suitable patients who had been diagnosed with renal cell carcinoma, and for whom surgical removal of the diseased tissue was deemed necessary, were identified by the urological surgical team (P.K., N.R., C.H.). Written informed consent for human tissue procurement was obtained from all participants following their consenting to the surgical procedure; information sheets documenting the proposed use of tissues was provided by the urological team during a clinical consultation. Ethical approval for the study was provided by The Office for Research Ethics Committees Northern Ireland (ORECNI; 07/NIR02/102). Once adequate tissue was obtained for histological diagnosis and staging of the tumor, tumor-associated vascular tissue was identified and removed from the specimen by the consultant pathologist (D.O’R.) and placed in ice-cold Krebs’ as previously reported [15]. Following this, pharmacological characterization of rucaparib was performed as for rat aorta. Two segments of artery were assayed per patient, with one exception; in the case of one patient’s tissue, one tissue segment was constricted using PE as normal, and rucaparib effects were determined; the second segment contracted spontaneously in an oscillatory manner, meaning constriction with PE was unnecessary; in this instance, the impact of rucaparib on the magnitude of these oscillatory constrictions was assessed. For this patient, only a single concentration of rucaparib was investigated (100 μM).

## Results

### Pharmacological assessment of rucaparib

PE (10 μM) consistently evoked sub-maximally attainable constrictions in both tail artery and aortic sections. 10 μM PE constricts both rat arterial tissues to approximately 50% of what is achievable. This level of constriction allows for characterization of the effects of agents on vascular tone, either dilatory or constrictory. Although constrictions evoked by 10 μM PE are below the maximal degree achievable, they were assigned a value of 100%, and subsequent changes in vessel tone were expressed relative to 10 μM PE.

**Fig 1:**
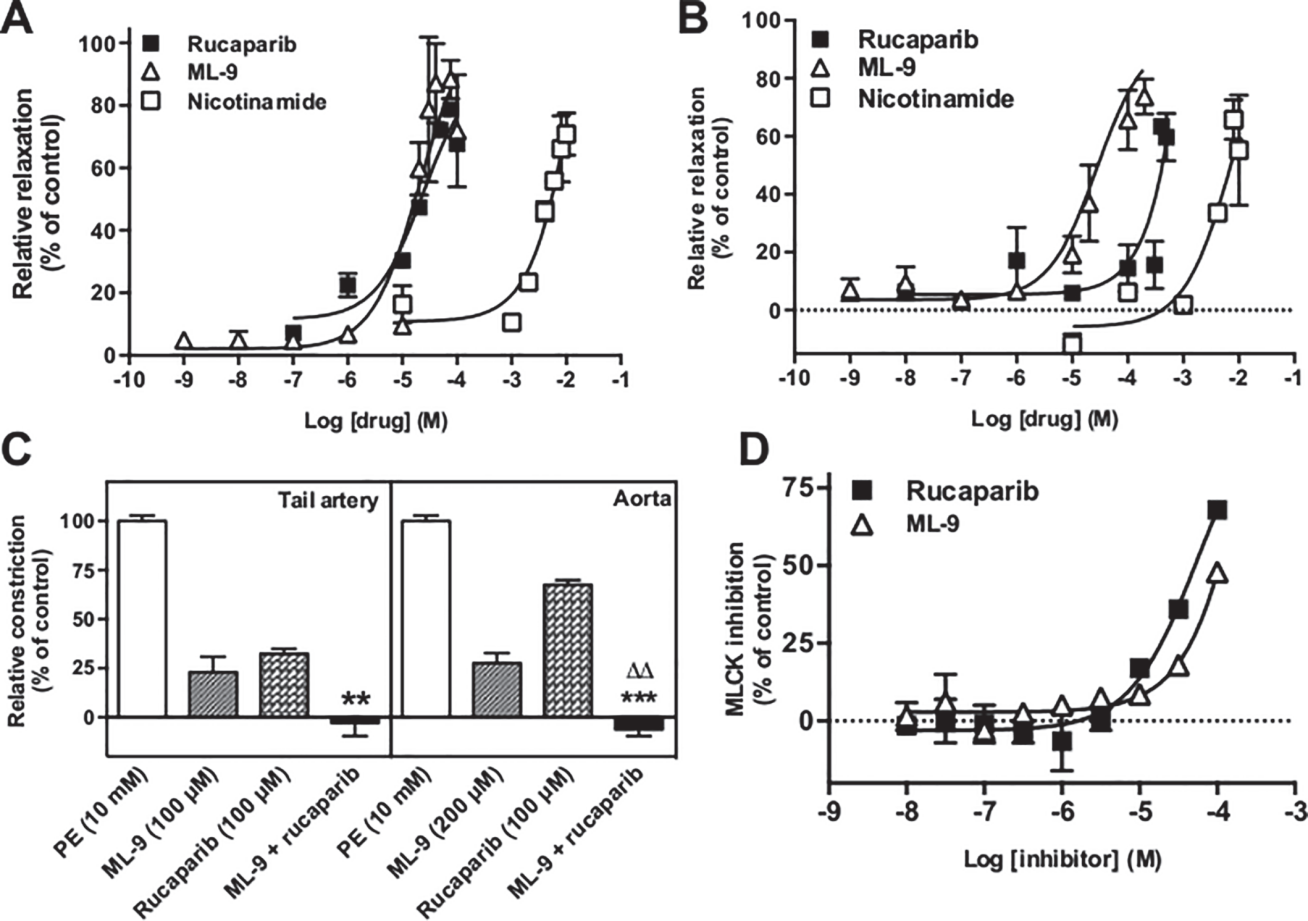
Rucaparib-mediated vasodilation of rat vascular tissue may be partially dependent on myosin light chain kinase. Panel A; rucaparib (closed squares), nicotinamide (open squares) and ML-9 (open triangles) inhibit smooth muscle contraction in PE-constricted rat tail artery. Artery sections were constricted using 10 μM PE before perfusion with a solution containing 10 μM PE plus the relevant concentration of drug. Panel B; rucaparib, nicotinamide and ML-9 inhibit smooth muscle contraction in PE-constricted rat aorta. Panel C; inhibition of arterial smooth muscle contraction by rucaparib is dependent on a mechanism in addition to MLCK inhibition. Constricted vessel segments were relaxed to the maximal degree achievable with ML-9, before being challenged with a relaxing cocktail of ML-9 plus rucaparib. The histograms illustrate the additive effects that were observed in the cases of both tail artery (left) and aorta (right). ** p<0.01, *** p<0.001 versus relaxation evoked by rucaparib alone; ^ΔΔ^ p<0.01 versus ML-9 alone. Bars represent mean of at least three independent experiments. Arteries from at least three rats were used per test. Error bars represent SEM. Panel D; rucaparib (closed squares) inhibits MLCK activity with ten times the potency of ML-9 (open triangles). Kinase activity was analyzed using the Millipore IC_50_ *Profiler* Express service. Points represent results of duplicate experiments. Error bars represent SEM.

Rucaparib concentration-dependently inhibited PE-induced constriction in rat tail artery segments (Fig. 1A; EC_50_ 23.7 μM). The MLCK inhibitor ML-9 elicited dilation with an EC_50_ of 20.1 μM, while the EC_50_ for nicotinamide was 23.7 mM. PE-constricted rat aorta also relaxed in response to rucaparib (Fig. 1B; EC_50_ 523 μM), ML-9 (EC_50_ 28 μM) and nicotinamide (EC_50_ 7.8 mM).

NO˙ is a potent vasodilator. To address whether rucaparib-evoked dilation was dependent on generation of NO˙, artery sections were pre-perfused with the NO˙ synthase (NOS) inhibitor, L-NMMA before challenge with rucaparib. Pre-perfusion of tail artery segments with L-NMMA had no significant effect on rucaparib-evoked dilation except at 100 μM, when it attenuated the rucaparib response from 68.84 ± 1.7% to 43.52 ± 4.98% relative dilation in tail artery (p<0.001) (S1A Fig.). L-NMMA did not significantly affect rucaparib-evoked dilation of aorta at any concentration tested (S1B Fig.). Acetylcholine (10 μM) elicited relaxation of PE-constricted tail artery and aorta segments (25.87 ± 3.3% and 14.86 ± 2.89% respectively), indicating endothelium (NO˙’s site of vasodilation induction) was intact (not shown).

### Potency of rucaparib-evoked inhibition of MLCK

In both arterial models, ML-9 relaxed tissues after PE constriction. Relaxation of PE-constricted segments with the maximally-relaxing concentration of ML-9 (100 μM in tail artery and 200 μM in aorta) followed by a combination of this concentration of ML-9 with 100 μM rucaparib resulted in additional relaxation of the arterial segments, returning the tone of the vessels to basal levels (Fig. 1C). In each case, dilation with a combination of the maximally active concentration of ML-9 plus 100 μM rucaparib was significantly greater than dilation elicited by 100 μM rucaparib alone (p = 0.009 in tail artery; p<0.001 in aorta). These data indicate that MLCK inhibition is not solely responsible for rucaparib’s vasoactivity.

In our previous study, we demonstrated that rucaparib inhibited the activity of MLCK in an *in vitro* assay [11]. The purpose of this experiment was to determine the potency of rucaparib relative to that of ML-9, a commercially available MLCK inhibitor. Although neither inhibitor evoked maximal inhibition of MLCK in the concentration range tested (10 nM—100 μM), rucaparib was ten times more potent an inhibitor of MLCK activity than ML-9 (IC_50_s of 55 μM [11] and 560 μM respectively; Fig. 1D).

### Determination of P2 receptor involvement in rucaparib-evoked relaxation

P2 receptors have been implicated in maintenance of vascular tone [16]. They are responsible for ATP-induced actions [17], and are either ATP-gated ion channels (P2X) or G-protein-coupled (P2Y) [18] receptor types. The agonists of these nucleotide receptors are ATP, ADP, UTP, UDP and NAD^+^. β-NAD^+^ was recently revealed to inhibit contraction of murine smooth muscle by its action at the P2Y_1_ receptor [19]. This, coupled with structural similarity of rucaparib to NAD^+^ led us to investigate the role of P2 receptors in rucaparib-evoked vasodilation.

**Fig 2:**
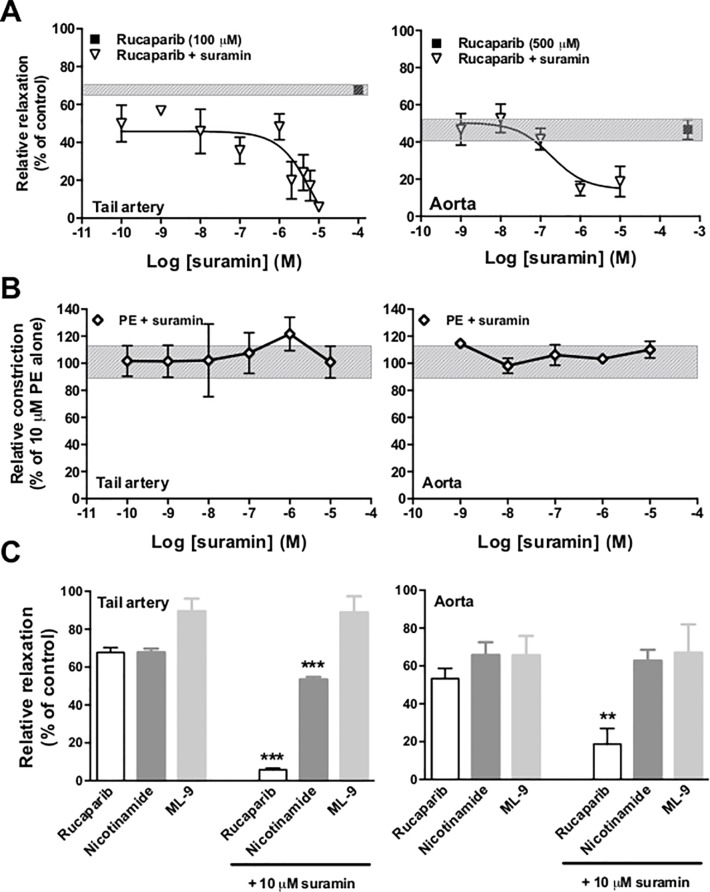
P2 receptor blockade abrogates rucaparib-evoked vasodilation. Panel A; broad-spectrum antagonism of P2 receptors using suramin (inverted open triangles) abrogated rucaparib-evoked relaxation in rat tail artery (left) and aorta (right). Panel B; suramin itself was without vasoactivity, demonstrable by its failure to alter the tone of sub-maximally constricted vessels. The shaded region represents the degree of constriction evoked using 10 μM PE alone. Panel C; suramin has little effect on vasodilation elicited by nicotinamide and none on vasodilation elicited by ML-9, but inhibits that elicited by rucaparib in tail artery and aorta. **p<0.01, ***p<0.001 as compared with dilation achieved in the absence of suramin. Arteries from at least three rats were used per test.

Perfusion of PE-constricted rat tail artery and aorta sections with suramin (P2 receptor antagonist; 100 pM—10 μM) concentration-dependently abrogated rucaparib-dependent relaxation (Fig. 2A). Suramin itself was devoid of any vasoactivity in both models (Fig. 2B). Suramin is not a universal inhibitor of smooth muscle relaxation, demonstrable by its inability to prevent relaxation evoked by ML-9 in either arterial model. In the tail artery model, suramin exerted a slight inhibition of nicotinamide-evoked dilation, but was without effect in the aorta model (Fig. 2C). As above (Fig. 2A), suramin potently blocked the effects of rucaparib in both arterial models.

**Fig 3:**
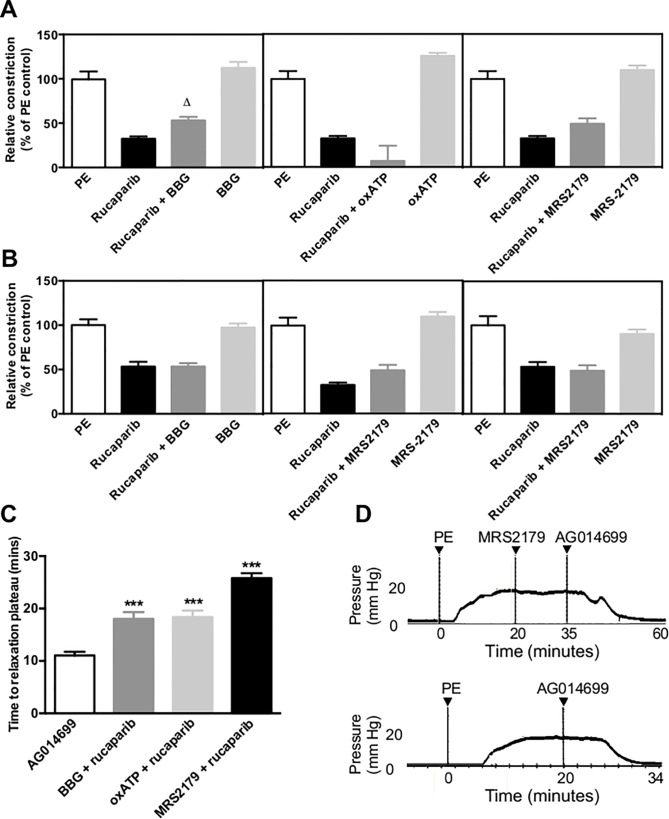
Rucaparib may act at multiple P2 receptor subtypes. Panels A and B; blockade of specific P2 receptor subtypes P2X_4_, P2X_7_ and P2Y_1_ in tail artery (A) and aorta (B) models failed to categorically identify the receptor subtype at which rucaparib elicits its dilatory effect. ^Δ^ p<0.05 as compared with the degree of dilation achieved by rucaparib in the absence of P2 antagonism. Panel C; summary of the duration of perfusion of tail artery segments necessary for relaxation plateau to be reached. Although the absolute degree of relaxation achieved was similar in the absence and presence of specific P2 receptor antagonism, the time taken for relaxation to complete was prolonged in all cases tested in tail artery. ***p<0.001 compared to time taken for relaxation plateau in the absence of P2 antagonism. Panel D; representative trace of rucaparib-induced relaxation when the P2Y_1_ receptor was antagonised using MRS-2179 (top), and rucaparib-induced relaxation (bottom). Bars represent mean of at least three independent experiments. Error bars represent SEM. Arteries from at least three rats were used per test.

Antagonism of specific P2 receptor subtypes P2X_4_, P2X_7_ and P2Y_1_ was unable to categorically identify the receptor responsible for the activity of rucaparib in arterial tissue. Antagonism of P2X_4_ with Brilliant Blue G in tail artery significantly lessened relaxation, although was without effect in aortic tissue (Fig. 3A and B). Neither P2X_7_ nor P2Y_1_ antagonism with oxidised ATP or MRS-2179 respectively affected the degree of relaxation elicited by rucaparib. Nevertheless, while the degree of relaxation achieved remained largely unchanged, Brilliant Blue G, oxidised ATP and MRS-2179 all significantly delayed the completion of rucaparib-evoked relaxation (determined as stable tone that did not vary (positively or negatively) for a duration of 20 s), compared with unobstructed relaxation (Fig. 3C and D).

## Role of PARP in rucaparib-mediated vasodilation

**Fig 4:**
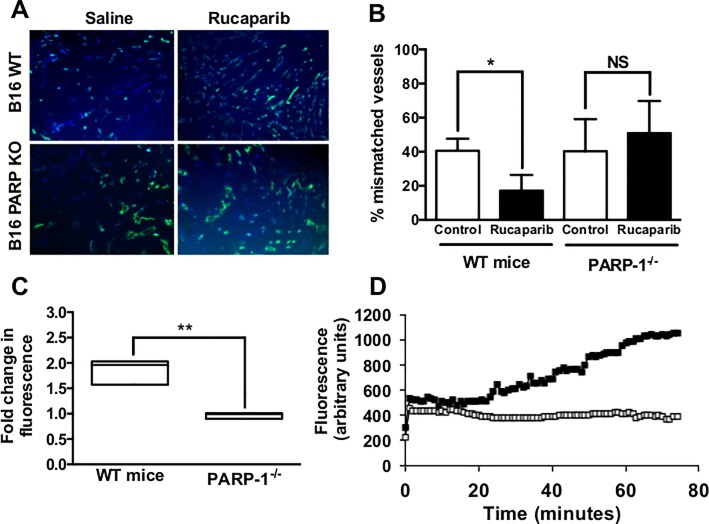
Effects of rucaparib on tumor vessel perfusion may be dependent on PARP. Panels A and B; B16 tumours were established in PARP WT or KO female mice. The extent of vessel ‘mismatch’ following the administration of the perfusion markers Hoechst 33342 and carbocyanin was reduced by rucaparib (1 mg/kg) in tumors established in WT but not PARP-1^-/-^ mice. Panel C; fold change in intratumoral fluorescence above that seen following initial plateau (20 min) in dorsal window chambers implanted with B16 tumors in WT and PARP-1^-/-^ mice and treated with 1 mg/kg rucaparib. Panel D; representative real time analysis of the accumulation of BSA-647 (administered via iv injection at time 0) in B16 tumors established in dorsal window chambers in PARP WT (closed symbols) and PARP-1^-/-^ (open symbols) mice. Arrow indicates the administration of rucaparib (10 mg/kg). NS—p >0.05, *p<0.05, **p<0.01 as compared with relevant control. N = 3 mice per condition.

A role for PARP itself in rucaparib’s vasoactivity was investigated using tumor vessel perfusion mismatch and dorsal window chamber analyses in C57BL/6 WT and PARP-1^-/-^ mice. Vessel mismatch studies work on the basis that, after venous delivery of two fluorescent dyes with an interval of 20 minutes between, vessels that are positive for both dyes are deemed to be ‘matched’, while those vessels that are positive for either dye alone are ‘mismatched’, a phenomenon that occurs by vessel opening or closing before or after fluorescent dye delivery. In WT mice treated with saline, 40.5 ± 7.14% of vessels were mismatched, while those treated with rucaparib had 17.1 ± 9.26% mismatched vessels (p = 0.026). Conversely, rucaparib treatment did not affect the degree of vessel mismatch in PARP-1^-/-^ mice; vessel mismatch in saline-treated mice was 40.3 ± 18.8%, while in rucaparib-treated mice, vessel mismatch was 50.9 ± 18.9% (p = 0.53; Fig. 4A and B), indicating that the PARP inhibitor lacked potency in PARP null vessels. This observation was supported by real-time monitoring of the accumulation of BSA-647 in B16 tumors in dorsal window chambers, when treatment with rucaparib in WT mice evoked impressive accumulation of fluorescence in tumors (1.85-fold increase in fluorescence above initial baseline in WT, 0.97-fold in PARP-1^-/-^; p = 0.004—Fig. 4C), while levels of BSA-647 following similar treatment in PARP-1^-/-^ mice failed to increase above baseline plateau (exemplar of one mouse from both groups can be seen in Fig. 4D).

### Vasoactivity of rucaparib in human tumor-associated vessels

**Fig 5:**
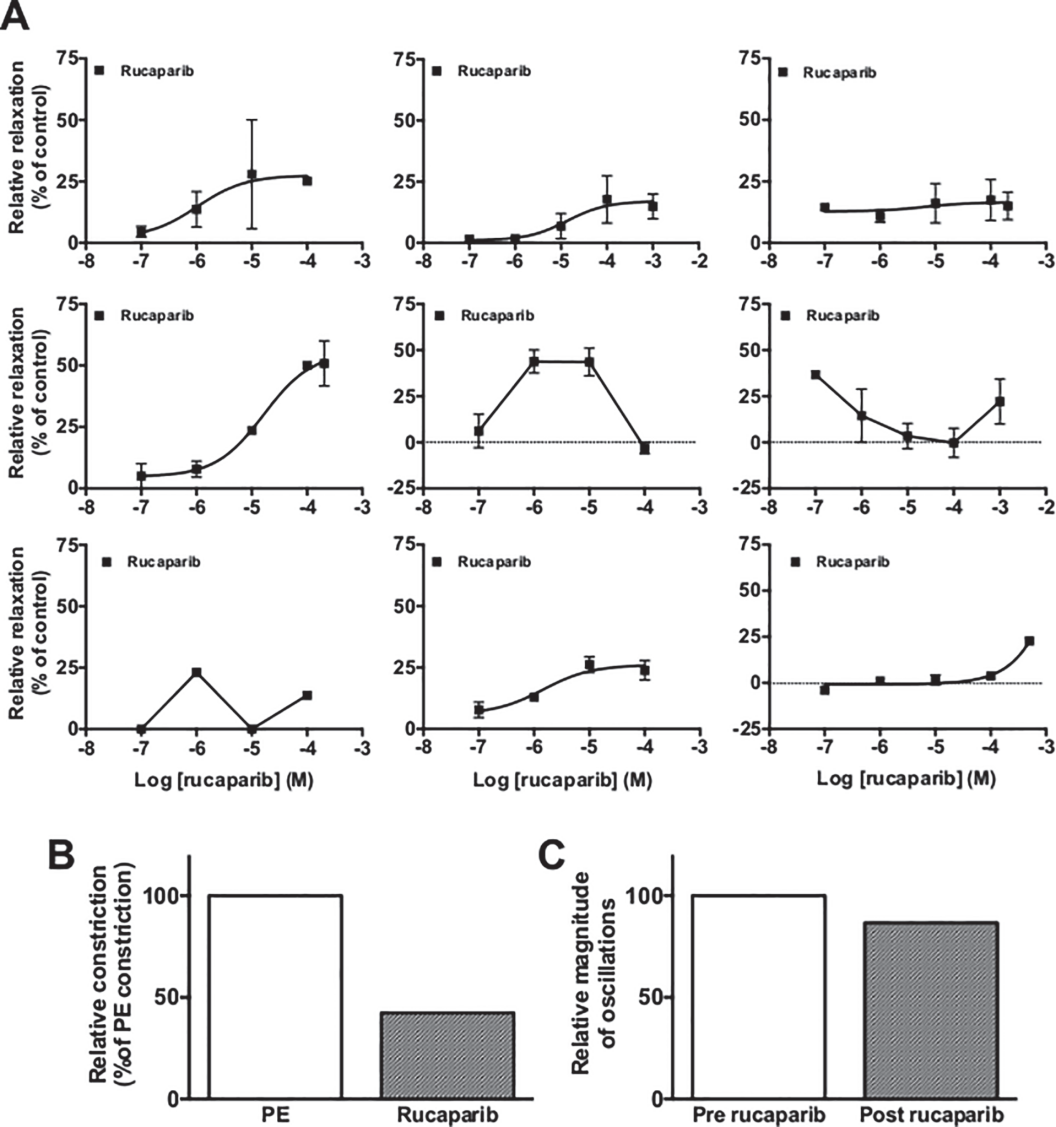
Heterogeneity of rucaparib activity in arteries isolated from patients having undergone nephrectomy. Panel A; concentration-response curves that were generated. Tissues were constricted using 10 μM PE, before being treated with PE and the relevant concentration of rucaparib. Panels B1 and B2; responses of two separate arterial sections from a single donor; panel B1 details the relaxant activity when a single tissue segment was constricted using 10 μM PE before being relaxed using 100 μM rucaparib; panel B2 details the slight inhibition of spontaneous oscillation of a single tissue segment that was observed following treatment with 100 μM rucaparib (the tissue contracted spontaneously, so no PE constriction was performed). Points/bars in most cases represent mean of two parallel experiments. Error bars represent SEM. Panels B1 and B2 error bars, as only a single observation was made per tissue section.

Previously, we investigated the vasoactivity of nicotinamide in vascular tissue isolated from patients post-nephrectomy for renal cell carcinoma [15], and found that nicotinamide blocked both spontaneous and phenylephrine-induced contraction, although not all vessels were responsive. In this study, we aimed to determine the pattern of response to rucaparib in similar tissues. In total, ten patients (all with renal cell carcinoma) consented to provide their tissue for inclusion in this study. From these, nine full concentration-response curves were constructed in tissues that were pre-constricted with 10 μM PE (Fig. 5A). There was heterogeneity of response that did not seem to be dependent on the gender of the tissue donor. The tissue summarised in panels B1 and B2 came from the tenth donor. Tissue section B1 was constricted with PE as normal and treated with 100 μM rucaparib. Tissue in panel B2 was not constricted with PE as the tissue contracted spontaneously in an oscillatory fashion; the data in panel B2 represents the effect of 100 μM rucaparib on the magnitude of these spontaneous oscillations (Fig. 5B). The magnitude of responses of the tissues to PE are included in Table 1 to demonstrate that lack of response to rucaparib was not a result of poor contractility of the tissues, and also serves to demonstrate further heterogeneity in terms of degree of response of the tissues to the α_1_ agonist itself.

**Table 1:** Summary of rucaparib activity in PE-constricted human tumor-associated vasculature.

**Specimen**	**Gender of donor**	**Mean PE response (g)**	**Mean rucaparib relaxation (g)**
A1	Male	0.46	0.12
A2	Male	0.51	0.07
A3	Female	0.58	0.09
A4	Male	0.08	0.04
A5^a^	Male	0.83	0.37
A6	Male	2.47	0.78
A7	Male	0.58	0.08
A8	Female	1.76	0.39
A9	Male	1.71	0.39
B1^b^	Male	1.30	0.75

^a^Data presented represents the magnitude of vessel relaxation in response to the top concentration of rucaparib tested, except A5, where the second highest concentration is summarised.

^b^Vessel section B1 was constricted with PE as the ‘A’ vessels; B2 contracted spontaneously, so is not summarized in Table 1.

## Discussion

Vasoactive effects of PARP-1 inhibitors have been reported previously. In aging Fischer rats that showed impaired vasorelaxation, treatment with the PARP inhibitor INO-1001 improved maximal relaxation in response to acetylcholine [20], and PD128763 induced hypothermia (an indicator of hypotension) in mice [21]. The latter was an ‘off target’ vasoactivity effect, as the hypothermia following PD128763 treatment was observed in PARP-1^-/-^ mice as well as PARP-1^+/+^ mice. AG14361 provoked no such hypothermia in the same models [21].

It has been known for some time that the nicotinamide pharmacophore-containing PARP inhibitors may inhibit other NAD^+^-dependent pathways. Indeed, the early benzamides inhibited several metabolic pathways [22]. The more potent PARP-1 inhibitors in clinical development are likely to be less promiscuous, although they do inhibit other PARP enzymes to varying degrees. Indeed, rucaparib inhibits not only PARP-1, but also PARPs 2–4, and tankyrase 1 [23]. Rucaparib was more potent an inhibitor of MLCK activity than nicotinamide [10], and was ten times more potent than ML-9, (Fig. 1D), and was also a more potent vasodilator than ML-9, a known vasodilator [24]. Hence, a role for MLCK inhibition in rucaparib-evoked vasodilation is likely. Rucaparib and ML-9 were equipotent dilators in tail artery, while rucaparib was more potent than ML-9 in aorta, which could be due to differential expression or activity of MLCK between the two tissues [25]. NO˙ is a potent vasodilator (increases PKG activity, in turn phosphorylating MLCK, deactivating it [26]) that has been implicated in PARP inhibitor-mediated vasoactivity [27,28]. Although L-NMMA treatment did not significantly affect rucaparib-evoked dilation, interaction of rucaparib with other targets could account for the persistence of dilation when NOS enzymes were inhibited.

While the apparent absence of vasoactivity in PARP-1^-/-^ mice was curious (Fig. 4), the phenomenon is not without precedent. Inhibition of PARP-1 with veliparib sensitized wild-type murine embryonic fibroblasts to camptothecin, although PARP-1^-/-^ counterparts were not similarly sensitive, suggesting that PARP-1 inhibition and PARP-1 absence are not equivalent [29]. Similarly, PARP inhibition with olaparib manifested DNA damage accumulation and cell cycle arrest in wild-type DT40 cells, but PARP-1^-/-^ counterparts did not display these characteristics, and olaparib sensitized wild-type cells to methyl methanesulfonate, sensitivity that was lacking in PARP-1^-/-^ [30]. In that respect, our vasodilation results are comparable with the above, where inhibited PARP and absence of PARP did not correlate. PARP-1^-/-^ mice still retain PARPs 2–4 (which rucaparib inhibits [23]), although these PARPs were unable to compensate for PARP-1’s absence in PARP-1^-/-^ mice. Veliparib [29], olaparib and niraparib [30], BMN 673 and rucaparib [31] all trap PARP-1 at sites of DNA lesions, forming a putative toxic protein product. A similar scenario may exist in vascular muscle contraction, when a complex between rucaparib and PARP-1 is responsible for dilation, meaning rucaparib is impotent in PARP-1’s absence.

Structural similarity to NAD^+^ led us to hypothesize that rucaparib might interact with a P2 receptor, known to be responsible for β-NAD-mediated inhibition of smooth muscle contraction [19]. We were unable to categorically identify P2 receptors involved in vasoactivity, although P2X_1_, P2X_2_, P2Y_1_, P2Y_2_, P2Y_4_ and P2Y_6_ are all candidates [16]. Differential receptor expression may explain rucaparib’s higher potency (55-times) in tail artery over aorta. It was an interesting finding that P2 antagonism completely abolished rucaparib’s vasoactivity, as MLCK inhibition could have been expected to compensate for the impact of P2 blockade. NAD^+^ enters NAD^+^-deprived astrocytes through P2X_7_-gated channels [32]; it may be that a similar mechanism of internalization exists for rucaparib, whereby MLCK inhibition-mediated dilation is contingent upon P2X receptor-mediated internalization of the PARP-1 inhibitor.

The behaviour of human vascular tissue reported here is consistent with the behaviour of similar tissue from our previous human vessel study [15]. Dilation of tumor-supplying vessels should improve tumor oxygenation, thereby improving radiotherapy, or increase anticancer drug delivery, as well as improving delivery of the PARP inhibitor itself to the tumor, with consequent increased intra-tumoral PARP inhibition. Chemopotentiation of temozolomide by rucaparib has already been reported, and was proposed to be resultant of vasoactivity of the drug [11]. Pharmacokinetic data indicate that the C_max_ of rucaparib at the chemosensitizing dose of 12 mg/m^2^ was between 1.3 and 1.7 μM in patients [33]. As a single agent, oral rucaparib (840 mg BID) resulted in steady state levels of around 9 μM with trough levels 2 μM at doses 240 mg BID [34]. Vasoactivity of rucaparib was observed at 1 μM in both rat arterial tissues and in six of the nine patient vessels for which concentration response curves were constructed, suggesting that the phenomenon observed *in vitro* and in mice may have relevance in patients, although, to our knowledge, this has not been determined.

If vasodilation is a common feature of PARP inhibitors containing the nicotinamide pharmacophore, it would broaden the range of chemotherapeutics beyond temozolomide and the topoisomerase I poisons that could be enhanced. Indeed, there are numerous clinical trials combining PARP inhibitors with paclitaxel, carboplatin, and gemcitabine currently underway, and the success of these trials may be more dependent on improved drug delivery rather than inhibition of DNA repair. Rucaparib did not potentiate doxorubicin, although doxorubicin-associated cardiotoxicity was abrogated by the PARP-1 inhibitor [35]. The therapeutic benefits of carboplatin and gemcitabine in patients with triple negative breast cancer were significantly enhanced by BSI-201 [36]; as BSI-201 has now been conclusively demonstrated to not inhibit PARP [37], it is possible that its nicotinamide pharmacophore affords it vasoactivity-dependent chemopotentiation. Olaparib also dilated constricted rat arteries *ex vivo* [38]. As olaparib and BSI-201, like rucaparib, contain nicotinamide pharmacophores, these agents may exert vasoactivity via a similar mechanism.

The current report provides tantalising evidence of the mechanisms underlying rucaparib-mediated vasodilation. It was interesting to note that rucaparib was vasoactive in normal rat tissue as well as tumor-recruited mouse and human vessels. Vasoactivity in the human tumor-associated vessels was especially meaningful, as it translates preliminary lab-based findings to clinically relevant material. Differential P2 receptor expression may explain the heterogeneity of response we observed in patient material, and profiling patients may allow selection for maximum benefit from PARP inhibitor-cytotoxic combination therapy in the future.

## Supporting Information

S1 ARRIVE Guidelines Checklist (PDF)

S1 Fig Rucaparib-evoked vasodilation persists when nitric oxide synthases are inhibited. Panels A and B; rucaparib-evoked dilation of tail artery (A) and aorta (B) sections occurs independently of nitric oxide generation. Tail artery and aorta sections were constricted with 10 μM PE before treatment with rucaparib plus the relevant concentration of L-NMMA (open circles). The shaded regions represent the degree of relaxation achieved when vessel segments were treated with 100 μM rucaparib in the absence of L-NMMA. Points represent mean of at least three independent experiments. Error bars represent SEM.
(TIFF)

## References

[1] Quenet D, El Ramy R, Schreiber V, Dantzer F. The role of poly(ADP-ribosyl)ation in epigenetic events. Int J Biochem Cell Biol 2009; 41: 60–65. 10.1016/j.biocel.2008.07.023 18775502

[2] Schreiber V, Dantzer F, Ame JC, de Murcia G. Poly(ADP-ribose): novel functions for an old molecule. Nat Rev Mol Cell Biol 2006; 7: 517–528. 16829982 10.1038/nrm1963

[3] Curtin NJ, Szabo C. Therapeutic applications of PARP inhibitors: anticancer therapy and beyond. Mol Aspects Med 2013; 34: 1217–1256. 10.1016/j.mam.2013.01.006 23370117 PMC3657315

[4] Skalitzky DJ, Marakovits JT, Maegley KA, Ekker A, Yu XH, Hostomsky Z, et al Tricyclic benzimidazoles as potent poly(ADP-ribose) polymerase-1 inhibitors. J Med Chem 2003; 46: 210–213. 12519059 10.1021/jm0255769

[5] Calabrese CR, Almassy R, Barton S, Batey MA, Calvert AH, Canan-Koch S, et al Anticancer chemosensitization and radiosensitization by the novel poly(ADP-ribose) polymerase-1 inhibitor AG14361. J Natl Cancer Inst 2004; 96: 56–67. 14709739 10.1093/jnci/djh005

[6] Veuger SJ, Curtin NJ, Richardson CJ, Smith GC, Durkacz BW. Radiosensitization and DNA repair inhibition by the combined use of novel inhibitors of DNA-dependent protein kinase and poly(ADP-ribose) polymerase-1. Cancer Res 2003; 63: 6008–6015. 14522929

[7] Thomas HD, Calabrese CR, Batey MA, Canan S, Hostomsky Z, Kyle S, et al Preclinical selection of a novel poly(ADP-ribose) polymerase inhibitor for clinical trial. Mol Cancer Ther 2007; 6: 945–956. 17363489 10.1158/1535-7163.MCT-06-0552

[8] Plummer R, Lorigan P, Evans J, Steven N, Middleton M, Wilson R, et al First and final report of a phase II study of the poly(ADP-ribose) polymerase (PARP) inhibitor, AG014699, in combination with temozolomide (TMZ) in patients with metastatic malignant melanoma (MM). J Clin Oncol 2006; 24: 8013–0.

[9] Horsman MR, Chaplin DJ, Brown JM. Tumor radiosensitization by nicotinamide: a result of improved perfusion and oxygenation. Radiat Res 1989; 118: 139–150. 2523079

[10] Ruddock MW, Hirst DG. Nicotinamide relaxes vascular smooth muscle by inhibiting myosin light chain kinase-dependent signaling pathways: implications for anticancer efficacy. Oncol Res 2004; 14: 483–489. 15559762 10.3727/0965040042380478

[11] Ali M, Telfer BA, McCrudden C, O'Rourke M, Thomas HD, Kamjoo M, et al Vasoactivity of AG014699, a clinically active small molecule inhibitor of poly(ADP-ribose) polymerase: a contributory factor to chemopotentiation in vivo? Clin Cancer Res 2009; 15: 6106–6112. 10.1158/1078-0432.CCR-09-0398 19789326 PMC2756456

[12] de Murcia JM, Niedergang C, Trucco C, Ricoul M, Dutrillaux B, Mark M, et al Requirement of poly(ADP-ribose) polymerase in recovery from DNA damage in mice and in cells. Proc Natl Acad Sci U S A 1997; 94: 7303–7307. 9207086 10.1073/pnas.94.14.7303 PMC23816

[13] Murray J, Thomas H, Berry P, Kyle S, Patterson M, Jones C, et al Tumour cell retention of rucaparib, sustained PARP inhibition and efficacy of weekly as well as daily schedules. Br J Cancer 2014; 110: 1977–1984. 10.1038/bjc.2014.91 24556618 PMC3992512

[14] Williams KJ, Telfer BA, Shannon AM, Babur M, Stratford IJ, Wedge SR. Combining radiotherapy with AZD2171, a potent inhibitor of vascular endothelial growth factor signaling: pathophysiologic effects and therapeutic benefit. Mol Cancer Ther 2007; 6: 599–606. 17308057 10.1158/1535-7163.MCT-06-0508

[15] Ruddock MW, Burns DM, McKeown SR, Murphy L, Walsh IK, Keane PF, et al Contractile properties of human renal cell carcinoma recruited arteries and their response to nicotinamide. Radiother Oncol 2000; 54: 179–184. 10699482 10.1016/s0167-8140(99)00163-2

[16] Wallace A, Knight GE, Cowen T, Burnstock G. Changes in purinergic signalling in developing and ageing rat tail artery: importance for temperature control. Neuropharmacology 2006; 50: 191–208. 16226282 10.1016/j.neuropharm.2005.08.019

[17] Dalziel HH, Westfall DP. Receptors for adenine nucleotides and nucleosides: subclassification, distribution, and molecular characterization. Pharmacol Rev 1994; 46: 449–466. 7899473

[18] Kunapuli SP, Daniel JL. P2 receptor subtypes in the cardiovascular system. Biochem J 1998; 336 (Pt 3): 513–523. 9841859 10.1042/bj3360513 PMC1219898

[19] Mutafova-Yambolieva VN, Hwang SJ, Hao X, Chen H, Zhu MX, Wood JD, et al Beta-nicotinamide adenine dinucleotide is an inhibitory neurotransmitter in visceral smooth muscle. Proc Natl Acad Sci U S A 2007; 104: 16359–16364. 17913880 10.1073/pnas.0705510104 PMC2042211

[20] Pacher P, Vaslin A, Benko R, Mabley JG, Liaudet L, Hasko G, et al A new, potent poly(ADP-ribose) polymerase inhibitor improves cardiac and vascular dysfunction associated with advanced aging. J Pharmacol Exp Ther 2004; 311: 485–491. 15213249 10.1124/jpet.104.069658 PMC2527587

[21] Calabrese CR, Batey MA, Thomas HD, Durkacz BW, Wang LZ, Kyle S, et al Identification of potent nontoxic poly(ADP-Ribose) polymerase-1 inhibitors: chemopotentiation and pharmacological studies. Clin Cancer Res 2003; 9: 2711–2718. 12855651

[22] Cleaver JE, Morgan WF. 3-Aminobenzamide, an inhibitor of poly(ADP-ribose) polymerase, is a stimulator, not an inhibitor, of DNA repair. Exp Cell Res 1987; 172: 258–264. 3115799 10.1016/0014-4827(87)90385-5

[23] Wahlberg E, Karlberg T, Kouznetsova E, Markova N, Macchiarulo A, Thorsell AG, et al Family-wide chemical profiling and structural analysis of PARP and tankyrase inhibitors. Nat Biotechnol 2012; 30: 283–288. 10.1038/nbt.2121 22343925

[24] Gong MC, Cohen P, Kitazawa T, Ikebe M, Masuo M, Somlyo AP, et al Myosin light chain phosphatase activities and the effects of phosphatase inhibitors in tonic and phasic smooth muscle. J Biol Chem 1992; 267: 14662–14668. 1321813

[25] Deng M, Ding W, Min X, Xia Y. MLCK-independent phosphorylation of MLC20 and its regulation by MAP kinase pathway in human bladder smooth muscle cells. Cytoskeleton (Hoboken) 2011; 68: 139–149. 10.1002/cm.20471 20722044 PMC5664925

[26] Lincoln TM, Dey N, Sellak H. Invited review: cGMP-dependent protein kinase signaling mechanisms in smooth muscle: from the regulation of tone to gene expression. J Appl Physiol (1985) 2001; 91: 1421–1430. 11509544 10.1152/jappl.2001.91.3.1421

[27] Soriano FG, Pacher P, Mabley J, Liaudet L, Szabo C. Rapid reversal of the diabetic endothelial dysfunction by pharmacological inhibition of poly(ADP-ribose) polymerase. Circ Res 2001; 89: 684–691. 11597991 10.1161/hh2001.097797

[28] English FA, McCarthy FP, Andersson IJ, Stanley JL, Davidge ST, Baker PN, et al Administration of the PARP inhibitor Pj34 ameliorates the impaired vascular function associated with eNOS(-/-) mice. Reprod Sci 2012; 19: 806–813. 10.1177/1933719111433885 22421449

[29] Patel AG, Flatten KS, Schneider PA, Dai NT, McDonald JS, Poirier GG, et al Enhanced killing of cancer cells by poly(ADP-ribose) polymerase inhibitors and topoisomerase I inhibitors reflects poisoning of both enzymes. J Biol Chem 2012; 287: 4198–4210. 10.1074/jbc.M111.296475 22158865 PMC3281688

[30] Murai J, Huang SY, Das BB, Renaud A, Zhang Y, Doroshow JH, et al Trapping of PARP1 and PARP2 by Clinical PARP Inhibitors. Cancer Res 2012; 72: 5588–5599. 10.1158/0008-5472.CAN-12-2753 23118055 PMC3528345

[31] Murai J, Huang SY, Renaud A, Zhang Y, Ji J, Takeda S, et al. Stereospecific PARP Trapping by BMN 673 and Comparison with Olaparib and Rucaparib. Mol Cancer Ther 2014. 10.1158/1535-7163.MCT-13-0803 PMC3946062 24356813

[32] Alano CC, Garnier P, Ying W, Higashi Y, Kauppinen TM, Swanson RA. NAD+ depletion is necessary and sufficient for poly(ADP-ribose) polymerase-1-mediated neuronal death. J Neurosci 2010; 30: 2967–2978. 10.1523/JNEUROSCI.5552-09.2010 20181594 PMC2864043

[33] Plummer R, Jones C, Middleton M, Wilson R, Evans J, Olsen A, et al Phase I study of the poly(ADP-ribose) polymerase inhibitor, AG014699, in combination with temozolomide in patients with advanced solid tumors. Clin Cancer Res 2008; 14: 7917–7923. 10.1158/1078-0432.CCR-08-1223 19047122 PMC2652879

[34] Shapiro G, Kristeleit R, Middleton M, Burris H, Molife LR, Evans J, et al Abstract A218: Pharmacokinetics of orally administered rucaparib in patients with advanced solid tumors. Molecular Cancer Therapeutics 2013; 12: A218–A218.

[35] Ali M, Kamjoo M, Thomas HD, Kyle S, Pavlovska I, Babur M, et al The clinically active PARP inhibitor AG014699 ameliorates cardiotoxicity but does not enhance the efficacy of doxorubicin, despite improving tumor perfusion and radiation response in mice. Mol Cancer Ther 2011; 10: 2320–2329. 10.1158/1535-7163.MCT-11-0356 21926192 PMC3242069

[36] Pal SK, Mortimer J. Triple-negative breast cancer: novel therapies and new directions. Maturitas 2009; 63: 269–274. 10.1016/j.maturitas.2009.06.010 19632796

[37] Patel AG, De Lorenzo SB, Flatten KS, Poirier GG, Kaufmann SH. Failure of iniparib to inhibit poly(ADP-Ribose) polymerase in vitro. Clin Cancer Res 2012; 18: 1655–1662. 10.1158/1078-0432.CCR-11-2890 22291137 PMC3306513

[38] Senra JM, Telfer BA, Cherry KE, McCrudden CM, Hirst DG, O'Connor MJ, et al Inhibition of PARP-1 by olaparib (AZD2281) increases the radiosensitivity of a lung tumor xenograft. Mol Cancer Ther 2011; 10: 1949–1958. 10.1158/1535-7163.MCT-11-0278 21825006 PMC3192032

