# Supplementary figures and images for: Vasoactivity of Rucaparib, a PARP-1 Inhibitor, is a Complex Process that Involves Myosin Light Chain Kinase, P2 Receptors, and PARP Itself
Source: PLoS One. 2015 Feb 17;10(2):e0118187. doi: 10.1371/journal.pone.0118187 (PMC4331495; doi:10.1371/journal.pone.0118187)

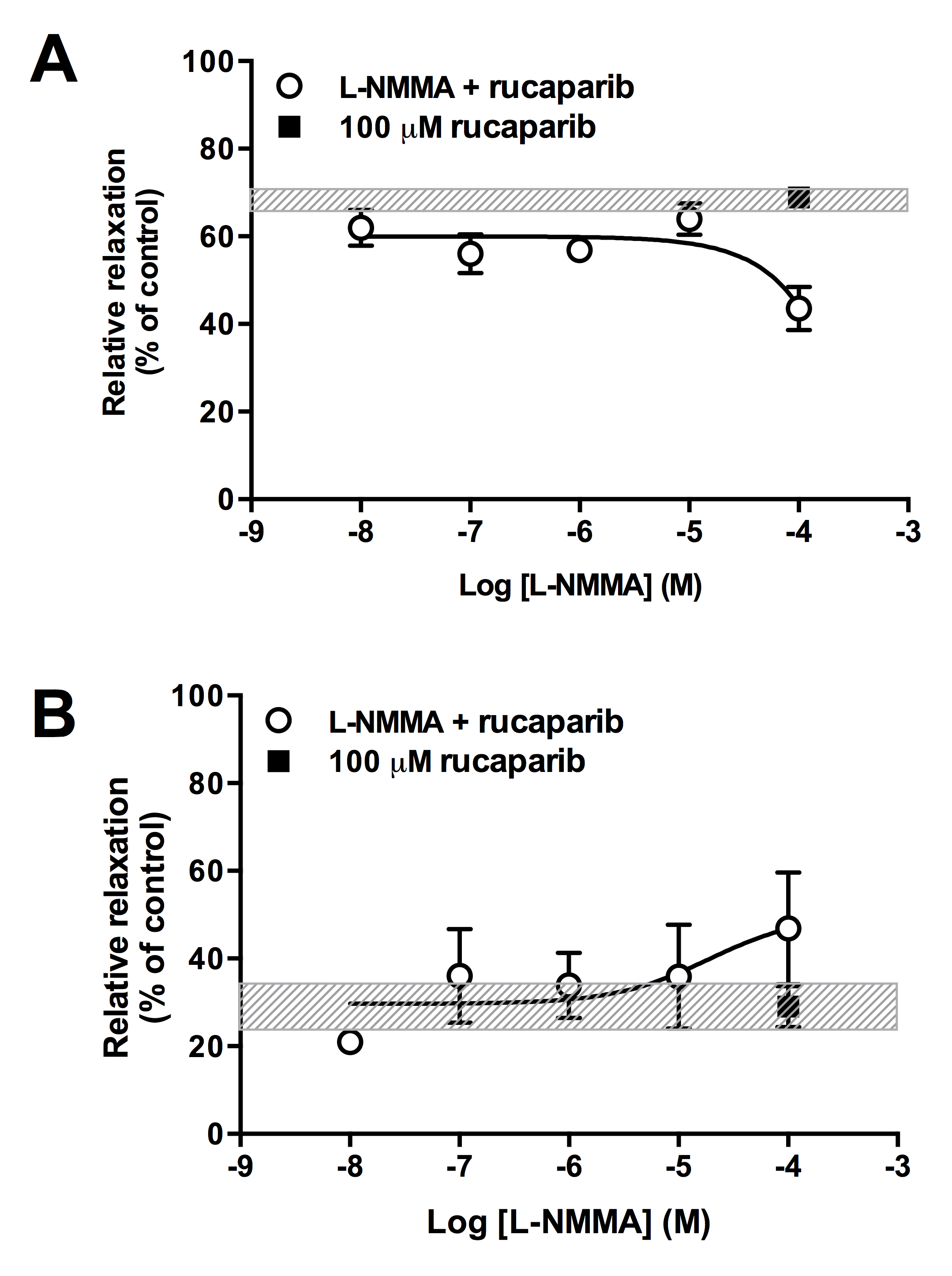

Supplement: S1 Fig — Panels A and B; rucaparib-evoked dilation of tail artery (A) and aorta (B) sections occurs independently of nitric oxide generation. Tail artery and aorta sections were constricted with 10 μM PE before treatment with rucaparib plus the relevant concentration of L-NMMA (open circles). The shaded regions represent the degree of relaxation achieved when vessel segments were treated with 100 μM rucaparib in the absence of L-NMMA. Points represent mean of at least three independent experiments. Error bars represent SEM. (TIFF) [file pone.0118187.s002.tiff]
